# Divergent changes in the elevational gradient of vegetation activities over the last 30 years

**DOI:** 10.1038/s41467-019-11035-w

**Published:** 2019-07-05

**Authors:** Mengdi Gao, Shilong Piao, Anping Chen, Hui Yang, Qiang Liu, Yongshuo H. Fu, Ivan A. Janssens

**Affiliations:** 1 Sino- French Institute for Earth System Science, College of Urban and Environmental Sciences, Peking University, 100871 Beijing, China; 2 Key Laboratory of Alpine Ecology, Institute of Tibetan Plateau Research, Center for Excellence in Tibetan Earth Science, Chinese Academy of Sciences, 100085 Beijing, China; 3 Department of Biology, Colorado State University, Fort Collins, CO 80523 USA; 4 College of Water Sciences, Beijing Normal University, Beijing, China; 5 Department of Biology, University of Antwerp, Universiteitsplein 1, B-2610 Wilrijk, Belgium

**Keywords:** Ecology, Ecology

## Abstract

The reported progressive change of vegetation activity along elevational gradients has important aesthetic and conservation values. With climate change, cooler locations are suggested to warm faster than warmer ones, raising concerns of a more homogenized landscape along the elevation. Here, we use global satellite data to investigate the spatio-temporal dynamics of the elevational gradient (EG) in vegetation greenness (NDVI_max3_), spring (SOS) and autumn phenology (EOS) during 1982–2015. Although we find clear geographical patterns of the EG in NDVI_max3_ and SOS, there are no prevalent trends of vegetation homogenization or phenology synchronization along elevational gradients. Possible mechanisms, including spatially heterogeneous temperature lapse rate changes, different vegetation sensitivities to climate change, and human disturbances, may play diverse roles across different regions. Our finding of mixed EG trends and no general rules controlling EG dynamics poses challenges for mitigating possible adverse impacts of climate change on mountainous biological diversity and ecosystem services.

## Abstract (web summary)

Climate change may lead to changes in elevational patterns of vegetation activitities. Here, the authors analyze global remotely sensing data collected during 1982–2015 to investigate the spatio-temporal dynamics of the elevational gradient in vegetation activities.

## Introduction

Vegetation activity often displays a clear elevational pattern^1,2^. Such an elevational pattern of vegetation activity can refer to vegetation phenology. For example, the dates of spring leaf unfolding and autumn leaf senescence have been observed to progressively change from low to high elevations for many regions^3,4^. It can also be related to vegetation growth and greenness^5^. At many places, vegetation growth and greenness have been found to change progressively with elevation as a result of differences in climate or in nutrient availability^6^ (relative to an optimum). Spatially, progressively changing vegetation activity along elevational gradients have been reported for many temperate and boreal regions^7^, with some cases also observed for the tropics^8,9^. This progressive vegetation activity change creates fascinating natural beauties that have been subjects of admirations in poems and proses for many generations. For instance, the great Chinese poet *Bai Juyi* once famously wrote, “In the plains past April, flowers have all but gone; In the hills at the temple, ‘tis the time for the peach blossoms to bloom”. It also helps maintain a diverse landscape of ecosystem structures and functions, particularly in mountainous areas that harbor the highest terrestrial biodiversity and provide critical ecosystem services to society.

Elevation-dependent temperature differences are often regarded as the main cause for the observed differences in vegetation activity along elevational gradients^5^. With climate change, however, there is an increasing concern that this elevationally progressive pattern of vegetation activity may be altered^10–12^. Specifically, the warming rate is often faster at higher than at lower elevations, leading to a trend of temperature homogenization^13^. This temperature homogenization has also been observed along latitudinal gradients, where it is believed to contributed to species and ecosystem homogenization^14^. The question thus arises whether, analogous to latitudinal temperature and ecosystem homogenization, elevation-dependent warming would also lead to more homogenized or synchronized vegetation activity? The elevational pattern of vegetation activity changes over the past decades under the reported significant climate change has to date not been studied. Because a more homogenized or synchronized vegetation pattern may reduce both the aesthetic aspects and functional diversities, addressing these questions is important for understanding and predicting vegetation activity and its ecosystem functioning under climate change.

Here, we use satellite-derived vegetation activity data to investigate how the relationship between vegetation activity and elevation may have changed over the past three decades. We consider three vegetation activity indicators that are derived from the Advanced Very High Resolution Radiometer (AVHRR) GIMMS NDVI_3g_ dataset over 1982–2015 at the spatial resolution of 8 km × 8 km to represent vegetation activity strength and phenology: the maximum three-monthly normalized difference vegetation index (NDVI_max3_), the start date of the growing season (SOS), and the end date of the growing season (EOS). These three vegetation activity indices provide key information for understanding vegetation growth, carbon cycles, and vegetation phenology, and have been found to show clear elevational patterns in many regions^15,16^. Here the relationship between vegetation activity and elevation is quantified as an elevational gradient (EG), which is defined as the average change of a vegetation activity index per 100 m increase in elevation. For each 8 km × 8 km grid cell, we estimate multi-year EG of vegetation activities by regressing the multi-year averaged vegetation activity indicators against its elevation in a 9 × 9 moving window centered at the focused grid cell (see Methods). Similarly, we also estimate EG for each year with annual vegetation activity data, and then derive the temporal EG trend using least squares linear regressions between annual EG and the year (see Methods). Note in this study, we use the average value from four different phenology derivative algorithms as the estimated SOS or EOS (see Methods). To test the robustness of the results, we also use other moving window sizes, i.e., 5 × 5, 7 × 7, 11 × 11, 13 × 13 (see Methods). The aim of this study is three-fold: (1) to investigate the EG of the above three vegetation activity indicators during 1982–2015 and their spatial patterns; (2) to test if the temporal evolution of these elevational gradients follows a hypothetic trend of increased homogenization or synchronization; and (3) to identify possible mechanisms underlying the observed spatio–temporal dynamics of vegetation activity EG.

## Results

### Multi-year averaged EG of vegetation activities

**Fig. 1:**
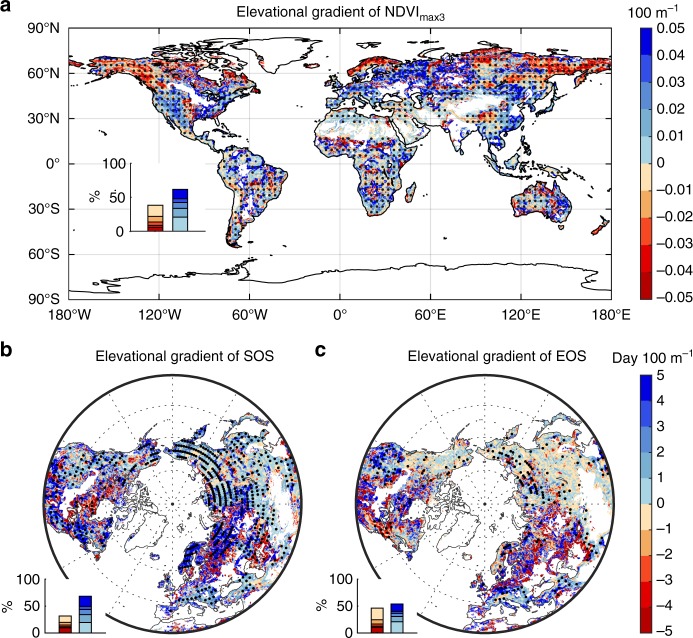
The elevational gradient (EG) of vegetation activity indices. **a** The spatial pattern of the EG of NDVI_max3_ (EG_ndvimax_) from 1982 to 2015. **b** the spatial pattern of the EG of SOS (EG_SOS_) from 1982–2011 over the Northern Hemisphere (north of 30°N). **c** The spatial pattern of the EG of EOS (EG_EOS_) from 1982 to 2011 over the Northern Hemisphere. A frequency distribution of the EG values is shown in the inset at the bottom-left for each panel. Regions marked with dots have statistically significant (*p* < 0.05 with the *F*-test) EG values

We first explored the spatial patterns of the multi-year averaged EG values for each of the three vegetation activity indices during 1982–2015 (Fig. 1). The results showed highly heterogeneous, rather than consistent patterns of EG values across the global land surface for all three vegetation activity indices. The EG of NDVI_max3_ (EG_ndvimax_) was negative (decreased vegetation greenness at higher elevations) in about 38% of the study area, mostly in cold regions including the Tibetan Plateau and the Artic regions (Fig. 1a). By contrast, it was positive (increased vegetation greenness at higher elevations) in the tropics and in most temperate regions, such as south-eastern America, Central Europe, and Eastern China (Fig. 1a). Furthermore, EG_ndvimax_ calculated with other moving window sizes also showed similar patterns (Supplementary Fig. 1), proving the robustness of the spatial pattern of EG_ndvimax_ shown in Fig. 1a.

**Fig. 2:**
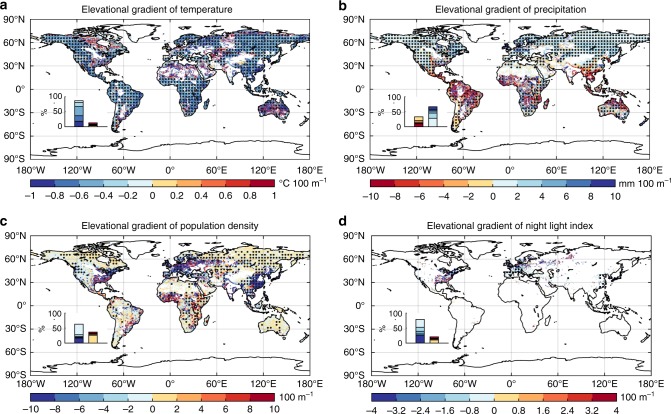
The elevational gradient (EG) of climate and human activity variables. **a** The spatial pattern of the EG of mean annual temperature (EG_tem_). **b** The spatial pattern of the EG of total precipitation (EG_pre_). **c** The spatial pattern of the EG of population density (EG_pd_). **d** The spatial pattern of the EG of night light index (EG_NLI_). A frequency distribution of the EG values is shown in the inset at the bottom-left for each panel. Regions marked with dots have statistically significant (*p* < 0.05 with the *F*-test) EG values

The highly varied EG_ndvimax_ pattern may be caused by regional differences in the dominant mechanisms governing vegetation greenness. In cold regions, vegetation growth is mostly limited by low temperature^17^, and decreased vegetation greenness with increasing elevation (negative EG_ndvimax_, Fig. 1a) is therefore probably related to the cooler temperature at higher elevations (negative EG in temperature (EG_tem_), Fig. 2a and Supplementary Fig. 2a). In the tropics, where vegetation often grows near its thermal optimum, temperature may not be such a dominant limiting factor for vegetation greenness^18,19^. Instead, in the tropics the lower temperatures at increasing elevation may actually reduce evapotranspiration demand and respiratory carbon losses, resulting in enhanced vegetation growth and greenness at higher elevations. Furthermore, in many temperate regions, the increase of precipitation (Fig. 2b and Supplementary Fig. 2b) may have contributed to the observed positive EG_ndvimax_ (Fig. 1a). Numerous field studies^20,21^ have suggested that increasing precipitation along the elevation facilitated vegetation growth at higher elevations. The positive EG_ndvimax_ may also be partly attributed to the reduction of human disturbances at higher elevations (Fig. 2c, d). For instance, the impact of reduced human activities at higher elevations, including reduced thinning intensity and less pollution^22^, can be particularly important for determining the sign of EG_ndvimax_ in densely populated regions like Eastern North America, Europe, Eastern and Southern China.

The spatial distribution of the elevation gradient of SOS (EG_SOS_) was also very heterogeneous, although positive EG_SOS_ (later start of growing season at higher elevations) dominated in most of the study area (about 68%, Fig. 1b). The largest EG_SOS_ appeared in Northern Europe and in south-eastern America, where SOS dates were often delayed by more than 5 days when climbing up every 100 m (Fig. 1b). By contrast, negative EG_SOS_ values were found in northeast North America and central Europe (Fig. 1b). In addition, analyses with each of the four methods in deriving SOS from satellite imagery (Supplementary Fig. 3), rather than using the four-method averaged value, all showed similar spatial patterns in EG_SOS_ as those shown in Fig. 1b. The corroborated results with different methods further confirmed the robustness of our finding about the spatial distribution of EG_SOS_ signs.

The positive EG_SOS_ over most regions is very likely attributable to the decreased temperature with elevation^3^, i.e. negative EG_tem_. As shown in Supplementary Fig. 4a and 4b, spring temperature decreases with increasing elevation in most regions. In northeast North America, however, both WorldClim^23^ and ERA Interim temperature datasets^24^ show increasing spring temperature with elevation (positive spring EG_tem_), which explains the observed negative EG_SOS_ there.

Unlike EG_SOS_, the EG of EOS (EG_EOS_) showed a more fragmented spatial pattern (Fig. 1c). Positive EG_EOS_ values (later autumn senescence at higher elevations) were found in southeast Europe and western North America, while negative ones (earlier autumn senescence at higher elevations) were found in high latitude regions and eastern Asia. Also in contrast to EG_SOS_, the spatial patterns of EG_EOS_ derived with different methods did not always corroborate (Supplementary Fig. 5). In particular, the pattern derived with the piecewise logistic method was significantly different from that obtained with the other three methods (i.e., HANTS, polyfit, and double logistic, see Methods). Further analyses showed that autumn NDVI increased with elevation in most regions (Supplementary Fig. 6), and that the spatial pattern of the elevational gradient of autumn NDVI (EG_ndviau_) was consistent with that of EG_EOS_ based on the algorithms HANTS, polyfit, and double logistic, being mostly (56%) positive across the study area. The evidence from EG_ndviau_ therefore appears to support the elevational EOS changes derived with the algorithms of HANTS, polyfit, and double logistic.

Like spring temperature, autumn EG_tem_ was also mostly negative (Supplementary Fig. 7a, c), inconsistent with the highly mixed spatial distribution of EG_EOS_. This spatial mismatch between autumn EG_tem_ and EG_EOS_ suggests that autumn temperature may not be the main environmental factor controlling autumn phenology. It has been suggested that radiation, precipitation, winter warming and even spring phenology^25–27^ can all impact autumn phenology. We find that autumn precipitation amplifies with elevation in most regions (Supplementary Fig. 7b, d), which may contribute to the postponed autumn phenology and enhanced autumn NDVI at higher elevations in some regions. Furthermore, the temperature sensitivity of autumn phenology is smaller than that of spring phenology^17^, which may also help explain the more fragmented spatial pattern of EG_EOS_. Nonetheless, so far our knowledge on the major mechanisms shaping the spatio–temporal pattern of autumn phenology remains very limited^28–30^.

### Temporal dynamics of EG of vegetation activities

**Fig. 3:**
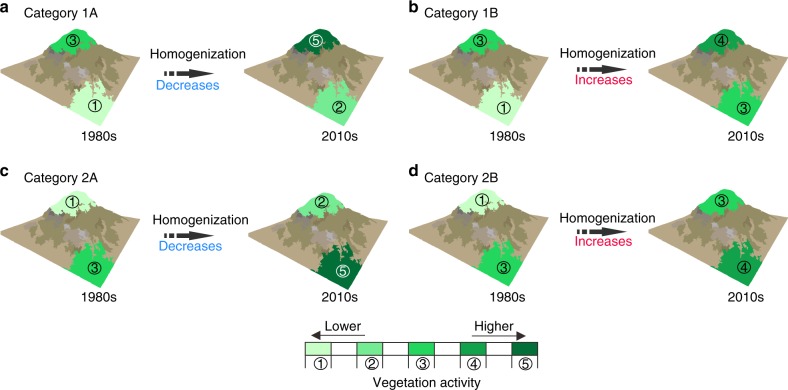
A diagram of changes in vegetation homogenization along elevational gradients. The vegetation activity here is showcased with the example of the maximum three-monthly normalized difference vegetation index (NDVI_max3_). Along the elevation, the multi-year averaged elevational gradient (EG) sign can be either (1) increasing vegetation activity (positive EG_ndvimax_) or (2) decreasing vegetation activity (negative EG_ndvimax_). Over time, the EG sign can either (A) amplified or (B) reduced. The combined changes along the elevation and over time for EG dynamics are illuminated in Fig. 3. Here (1A, **a**) and (2A, **c**) indicate decreased elevational vegetation homogenization; while (1B, **b**) and (2B, **d**) imply increased vegetation homogenization

Next, we examined the temporal trends of the annual EG of vegetation activities from 1982 to 2015. To facilitate hypothesis testing, we calculated for each grid cell both the multi-year averaged EG sign and the temporal change in EG. Along the elevational gradient, the multi-year averaged EG sign can either (1) indicate increasing vegetation activity (positive EG_ndvimax_, negative EG_SOS_, and positive EG_EOS_) or (2) suggest decreasing vegetation activity (negative EG_ndvimax_, positive EG_SOS_, and negative EG_EOS_). Over time, the EG sign can be either (A) amplified or (B) reduced. The combined changes in EG along the elevational gradient and over time are illuminated in Fig. 3. Here (1A) and (2A) indicate increased elevational vegetation dissimilarity; while (1B) and (2B) imply reduced vegetation dissimilarity, i.e. increased elevational vegetation homogenization/synchronization.

**Fig. 4:**
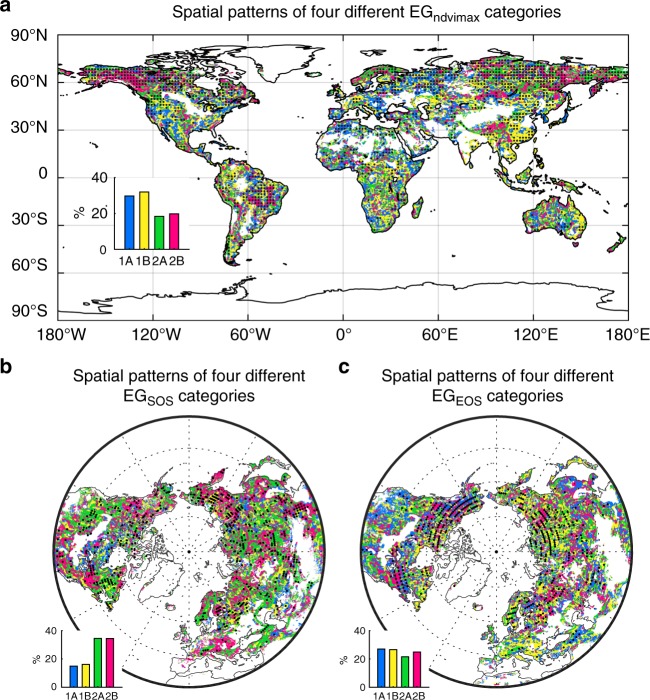
Spatial patterns of different temporal trends of the elevational gradient (EG) of vegetation activities. **a** The spatial pattern of different categories of the temporal trends in EG_ndvimax_ during 1982–2015. **b** The spatial pattern of different categories of the temporal trends in EG_SOS_ during 1982–2011 over the Northern Hemisphere (north of 30 °N). **c** The spatial pattern of different categories of the temporal trends in EG_EOS_ during 1982–2011 over the Northern Hemisphere (north of 30 °N). Category colors in this figure correspond to that shown in Fig. 3. Regions marked with dots have statistically significant (*p* < 0.05 with the *F*-test) EG values. The area fraction of each of the four different categories in the temporal trends of EG_ndvimax_/EG_SOS_/EG_EOS_ is shown in the inset at bottom-left for each panel

For vegetation greenness (EG_ndvimax_), we observed that about 48% of the study area falls into categories (1A) and (2A) (Fig. 4a). Spatially, the temporal trend of EG_ndvimax_ was very fragmented (Fig. 4a). Category (1A) was mainly found in southeast Europe, Eastern European highlands, southern Africa and the Rocky Mountains. By contrast, category (1B) was mostly observed in western India, Southeast China, eastern Australia, and western Europe. Category (2A) mainly occurred in the Siberian boreal region; while category (2B) was mainly found in Alaska and the Yukon region. Overall, this result of fragmented spatial distribution of the four categories of EG_ndvimax_ trends suggests that the elevational difference in vegetation greenness is not prevalently reduced and that the prediction of increased elevational homogenization of vegetation greenness is not supported.

The geographic distributions of the temporal trends in elevational temperature and precipitation gradients (EG_tem_ or the elevational lapse rate of temperature, and EG_pre_, respectively) were also mixed (Supplementary Fig. 8a, b). However, the spatial pattern of the sign of the EG_tem_ or EG_pre_ trends did not well match the pattern of the EG_ndvimax_ trends (comparing Fig. 4a with Supplementary Fig. 8a, b), indicating that changes in EG_tem_ or EG_pre_ may not have been the dominant reason for the observed EG_ndvimax_ trends at the global scale. Nonetheless, EG_tem_ or EG_pre_ changes may have explained some EG_ndvimax_ trends at the regional scale. For example, the reduced negative EG_ndvimax_ trend in western Europe may have resulted from the associated negative EG_tem_ trend, while the same EG_ndvimax_ trend in eastern Australia may be attributable to the negative EG_pre_ trend. Human activities may also have contributed considerably to the EG trends in some specific regions (Supplementary Fig. 8c, d). Fast population growth can imply increased anthropogenic disturbances and more frequent thinning (reduced vegetation greenness). For example, in central China, faster population growth at higher elevations may be responsible for the observed EG_ndvimax_ decrease, while in western Europe, faster population growth at lower elevations is likely one reason leading to the increased EG_ndvimax_ (Supplementary Fig. 8c, d). In addition, anthropogenic activities such as afforestation may also enhance vegetation activities. For instance, substantial afforestation has been implemented in regions like eastern China since the 1980s^31,32^, mainly at lower elevations (Supplementary Fig. 9). As a result, the increasing afforestation at lower elevations leads to a negative temporal trend of EG_ndvimax_.

We further investigated the changes of EG in spring and autumn phenology (SOS and EOS) using the same method. For both EG_SOS_ and EG_EOS_ we found that only about half of the study area fell into categories (1B) and (2B) that represent increased elevational synchronization of spring and autumn phenology (Fig. 4b, c). Therefore, these results also did not support the concept of global warming-induced synchronization at the global scale.

Interestingly, although most of the spatial pattern of EG_SOS_ could be explained by that of spring EG_tem_, the trend in spring EG_tem_ failed to explain the EG_SOS_ trend (Supplementary Fig. 10a, b). Further analyses suggested that the EG_SOS_ trend was also related to elevational gradient changes in the temperature sensitivity (St) of spring phenology (Supplementary Fig. 10c, see Methods). Based on the signs of the EG_SOS_ trend, the spring EG_tem_ trend, and the elevational gradient changes in St (EG_St_) of spring phenology, we also grouped all grids into another four categories according to the dominance of EG_tem_ and EG_St_ in controlling the EG_SOS_ trend (see Methods). We found that for about 31% of the Northern Hemisphere (NH) land area, the trend in EG_SOS_ was controlled by EG_St_, and also for about 31% of the NH, it was controlled by both EG_St_ and EG_tem_ (Supplementary Fig. 10d). On the other hand, in only about 20% of the area where the trend in EG_SOS_ was controlled by EG_tem_ (Supplementary Fig. 10d). Hence, the spatial pattern of EG_SOS_ trends was more dominated by changes in the temperature sensitivity of spring phenology instead of by changes in spring temperature. This important role of spring phenology sensitivity to temperature in shaping the spatial pattern of spring phenology was previously reported^33,34^.

## Discussion

We examined the spatio–temporal patterns of the elevational gradients of three vegetation activity indices representing vegetation greenness and phenology since 1982, and found a clear geographical pattern in the EG of vegetation greenness and spring phenology, but not in the EG of autumn phenology. Elevational temperature lapse was the dominant factor determining the elevational changes of spring phenology. Temperature was also important in shaping the EG of vegetation greenness; however, its significance and even the regulating mechanisms for the EG of vegetation greenness in tropical vs. boreal mountains were very different. The lack of explicit geographical pattern for the elevational gradient of autumn phenology and the spatial mismatch between the elevational gradients of autumn temperature and that of autumn phenology suggested that temperature may not be the main controlling factor for the EG of autumn phenology at the global scale. Little is known about the mechanisms controlling autumn phenology, especially when compared to our understanding of spring phenology. Further work on understanding mountainous autumn phenology is thus critically needed. Overall, our findings on the spatial patterns of the EG of vegetation activities suggest that temperature is not the sole dominant factor shaping the vegetation-elevation relationship, and highlights the importance of understanding vegetation activities and their drivers in the spatial context.

More importantly, we also found highly variable temporal changes for the elevational gradients of all vegetation activities (Fig. 4), in contrast to the hypothesized increased vegetation greenness homogenization or vegetation phenology synchronization along the elevational gradient in response to climate change. Furthermore, previous concerns of more homogenized/synchronized vegetation activities were based on the findings of enhanced warming rate at higher elevations^13,35–37^ and the assumption of the dominant role of temperature in determining vegetation activities^5,14,36^. Nonetheless, Zeng et al.^37^ suggested that warming is not always occurring faster at higher elevations. While vegetation activities can be more sensitive to climate change at higher elevations^3,5,38^, here we also find that mechanisms governing the spatio–temporal dynamics of the elevational gradients of vegetation greenness and phenology are not globally universal. Elevation-dependent warming did indeed lead to a reduction in the difference of spring phenology timing across the elevation gradient in some regions like the Alps^39^, yet temperature is not the sole element regulating the vegetation-elevation relationship^40,41^. Precipitation and human activities, for instance, have both played important roles in determining the elevational gradients of vegetation activities and their changes^40,42–45^, with various contributions in different areas. Furthermore, these multiple factors that control the vegetation-elevation relationship, as well as their recent dynamics, are highly heterogeneous across the global land surface. Such heterogeneities probably underpin the highly variable responses of the vegetation-elevation relationship to recent climate change. These variable vegetation-elevation responses under climate change need to be further investigated for better understanding and improved prediction of future vegetation activities under different climate scenarios.

Our results are subject to a few caveats. First, we used climate data from the Worldclim dataset, which was interpolated from meteorological station records. Considerable errors in deriving the gridded climate values in the dataset, particular in high latitude areas, may arise from the sparse distribution of meteorological stations. For example, the uniform pattern of EG_pre_ and EG_tem_ in high latitudes may be partly caused by the fact that climate in the surrounding grids was likely interpolated from the same station data (Fig. 2a, b). More meteorological stations are thus needed in data-sparse areas, especially at higher latitudes.

**Fig. 5:**
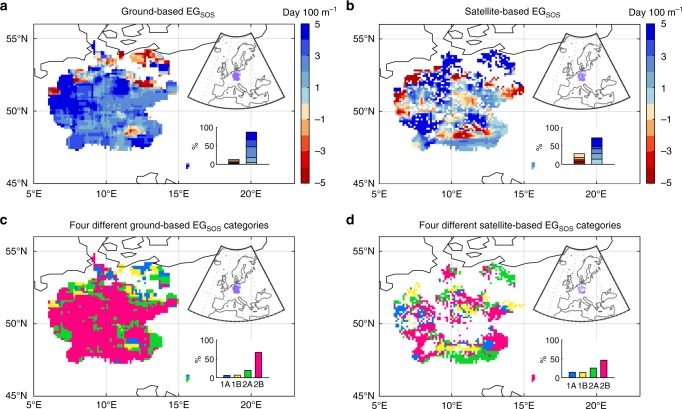
Spatial patterns of the elevational gradient of SOS (EG_SOS_) and their temporal trends. **a** The spatial pattern of ground-based EG_SOS_ from 1982 to 2011. **b** The spatial pattern of satellite-based EG_SOS_ from 1982 to 2011. Frequency distribution of the EG_SOS_ values is shown in the inset at the bottom-right for each panel. **c** The spatial pattern of different categories of the temporal trends in ground-based EG_SOS_ during 1982–2011. **d** The spatial pattern of different categories of the temporal trends in satellite-based EG_SOS_ during 1982–2011. The area fraction of each of the four different categories in the temporal trends of EG_SOS_ is shown in the inset at bottom-right for each panel. The location of the regions with European phenology stations is shown in the inset at top-right for each panel

**Fig. 6:**
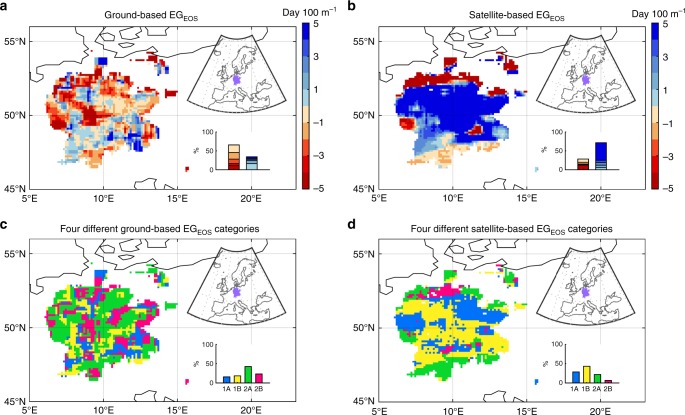
Spatial patterns of the elevational gradient of EOS (EG_EOS_) and their temporal trends. **a** The spatial pattern of ground-based EG_EOS_ from 1982 to 2011. **b** The spatial pattern of satellite-based EG_EOS_ from 1982 to 2011. Frequency distribution of the EG_EOS_ values is shown in the inset at the bottom-right for each panel. **c** The spatial pattern of different categories of the temporal trends in ground-based EG_EOS_ during 1982–2011. **d** The spatial pattern of different categories of the temporal trends in satellite-based EG_EOS_ during 1982–2011. The area fraction of each of the four different categories in the temporal trends of EG_EOS_ is shown in the inset at bottom-right for each panel. The location of the regions with European phenology stations is shown in the inset at top-right for each panel

Second, the GIMMS NDVI_3g_ dataset used in this study is at a spatial resolution of 8 km × 8 km, which can be rough for mountainous areas. However, this GIMMS NDVI_3g_ dataset is still likely the best one we can currently use for a global-scale analysis. For example, although the MODIS NDVI or EVI datasets has a higher spatial resolution, they cover a relatively short time period only since 2000. Future development of higher-resolution satellite datasets may further improve the robustness of such global-scale analyses of vegetation activity dynamics. To test the robustness of our satellite-based findings on the spatio–temporal EG trends of vegetation phenology, we perform the same analysis with a pan European phenological database PEP725, the largest ground-based phenological monitoring network. We find that the spatial pattern of ground-based EG_SOS_ is similar to that of satellite-based, i.e., later leaf unfolded date with increasing elevation (Fig. 5a, b); and the area fraction of each of the four different spatio–temporal EG trend categories for ground-based EG_SOS_ is also consistent with that of satellite-based EG_SOS_ (Fig. 5c, d). This again confirms the robustness of our finding based on large-scale satellite data analysis. However, for EOS, most regions show negative correlations between leaf coloration date and elevation (sooner start of leaf coloring at higher elevations) in station-based EG_EOS_, a pattern in weak agreement with that from satellite data (Fig. 6a, b). Such discrepancy between satellite- and ground-based EG_EOS_ spatial patterns in central Europe may partly arise from the sparse distribution of ground-based phenological stations, where only about 14.4 ± 5.8 sites have records for leaf unfold date and 12.1 ± 4.4 sites for leaf coloration for one 9 × 9 moving window. Furthermore, EOS from ground-based measurements is defined as the date with 50% leaf coloration, which may not be the same as satellite-based EOS derived from different algorithms detecting the abrupt change of NDVI. Nevertheless, the ground-based result still supports our major finding of no clear sign for increased elevational autumn phenology synchronization (Fig. 6c, d).

While our results do not support a prevalent trend of the elevational homogenization/synchronization of vegetation activities, such trends of recently increased homogenization/synchronization do appear in some regions (Fig. 4). Vegetation homogenization may have critical implications for landscape-scale diversity and ecosystem function^46^. For example, the increased vegetation phenological synchronization may reshuffle species composition and reduce species functional diversity^47^. Mountain areas harbor the most diverse terrestrial ecosystems and provide key ecosystem services, such as aesthetic appreciation, carbon storage, water conservation, and so on^48,49^. Increased vegetation homogenization and reduced species functional diversity along elevational gradients may impair the capacity of mountain ecosystems in providing these key services. Hence, for regions that do experience elevational vegetation homogenization, there is a great need to adopt appropriate policy tools to mitigate the adverse ecosystem impacts of such vegetation homogenization. These policy tools should be based on findings of region specific mechanisms driving the trend of vegetation homogenization.

Nonetheless, for most regions, we did not find apparent homogenization/synchronization trends in the EG of vegetation activities, nor do we have a clear answer on what drives them. More importantly, where a general rule governing the vegetation EG dynamics is lacking, even for regions where the vegetation EG trend was found to be statistically correlated with one or another environmental factor during the study period of 1982–2015, it remains highly uncertain how the vegetation EG will change in the future. This uncertainty imposes great challenges in predicting future landscape vegetation homogenization or phenology synchronization. It subsequently also casts uncertainty in best options for managing ecosystems to mitigate possible adverse impacts of climate change on mountainous biological diversity and ecosystem services.

## Methods

### Satellite-sensed vegetation index data

Normalized Difference Vegetation Index (NDVI) data is widely used as a proxy of vegetation greenness and photosynthetic activity^50,51^. Here we used the third generation Global Inventory Monitoring and Modeling Studies (GIMMS) group NDVI dataset^52^ (referred as NDVI_3g_). The dataset has a 15-day temporal frequency from 1982 to 2015 with a spatial resolution of 8 km. We removed cropland-dominated grids from the analysis for their intense human management. Specifically, grids with more than 80% coverage by croplands according to the MODIS MCD12C1 land cover classification^53^ were excluded. Note that this study excluded regions with bare soil/sparse vegetation where multi-year average NDVI is below 0.1.

### Climate dataset

Monthly average temperature and precipitation data used in this study was acquired from the Worldclim version 2 dataset^23^. The Worldclim dataset was generated by interpolating the average values of climatic records from 9000–60000 weather stations using thin-plate splines for the period 1970–2000, with a high spatial resolution of about 1 km × 1 km. We also used the reanalysis dataset ERA Interim dataset^24^ from the European Centre for Medium-Range Weather Forecasts (ECMWF), which provides monthly air temperature and precipitation data at a spatial resolution of 12 km for the period 1979–2018.

### Human activity dataset

The global datasets of population density and night light can represent the strength of human activities. Here the population density data were obtained from the Gridded Population of the World version 4 data (GPW v4) from the Socioeconomic Data and Applications Center at Columbia University. Population density for the years of 2000, 2005, 2010, and 2015 from GPW v4 was estimated at a spatial resolution of 1 km × 1 km, using an annualized growth rate extrapolation method based on the 2010 round of Population and Housing Censuses^54^. Night light data with a spatial resolution of 30 arc-seconds (approximately 1 km) for the period 1992–2013, covering regions from 65 °S to 75 °N, were downloaded from the Defense Meteorological Satellite Program—Operational Linescan System (DMSP-OLS) night light dataset^55^, which was derived from the National Oceanic and Atmospheric Administration (NOAA) National Geographic Data Center (NGDC).

### Ground-based phenology observation dataset

The ground-based phenology observation data are obtained across Central Europe from the Pan European Phenological database (PEP725). We seleted the records of the first leaf unfolding date and 50% leaf coloration date from 2256 sites and focused on the sites with continuous records for 1982–2011.

### Vegetation activity indicators

The maximum three-monthly NDVI_max3_ was calculated following the below steps: (i) calculating the mean monthly NDVI value for the period 1982–2015, and (ii) sorting the twelve average-monthly NDVI values to obtain the first three maximum values.

We also derived two phenological indices from the GIMMS NDVI_3g_ dataset, i.e. the SOS and the EOS. The SOS date was calculated as the average SOS date estimated by the below four methods: HANTS (harmonic analysis of time series), polyfit, timesat and spline^56–58^. The EOS date was obtained from the averaged EOS date estimated by the following four methods: HANTS, double logistic, piecewise logistic, and polyfit^15,57,59–61^.

### The calculation of elevation gradients

Before analyses, all datasets were resampled into a unified spatial resolution of 8 km (the same as the NDVI data). The elevation gradient of NDVI_max3_ (EG_ndvimax_) was calculated as the linear regression slope of the 34-year-averaged NDVI_max3_ against elevation for each 9 × 9 moving window. Digital Elevation Model (DEM) data^62^ was obtained from the Shuttle Radar Topographic Mission (SRTM) with a spatial resolution of 30 m. Similarly, the elevation gradient of SOS/EOS (EG_SOS_/EG_EOS_) was calculated as the linear regression slope of the multi-year-averaged of SOS/EOS against elevation within each 9 × 9 moving window. The elevation gradients of temperature (EG_tem_), total precipitation (EG_pre_), population density (EG_pd_), and night light index (EG_NLI_) were also calculated using the same methods. A positive elevation gradient indicates that the value of focused index is higher at higher elevations, while a negative one suggests that the value is higher at lower elevations. The statistical significance of the elevation gradient was determined by 95% confidence intervals. Note that only 9 × 9 windows with more than 40 grid cells of valid vegetation index values and more than 50 m in elevation difference were chosen for the regression analysis. Similar analyses were also performed using 5 × 5, 7 × 7, 11 × 11 or 13 × 13 moving windows to test the robustness of the results. Note that only 9 × 9 windows with more than 40 grid cells of valid vegetation index values and more than 50 meters in elevation difference were chosen for the regression analysis. Based on the Pan European Phenological database (PEP725), We calculate the elevation gradient of SOS (EOS) as the linear regression slope of leaf unfolded date (leaf coloration date) for each 9 × 9 moving window with more than five stations the same way as we do for satellite-based analyses.

### Driving factors of temporal trends in EG

To investigate possible effects of climate change and human activities on the temporal trends in vegetation activity EG, for each pixel we obtained the linear trends of EG in vegetation activity, climate, and night light index during 1982–2015 based on least-squares regressions. Note the data of night light index is available for 1982–2013; and because the number of available population density data for each grid during the study period is only 4, we therefore calculated the elevational gradient of the population density difference between 2015 and 2000 (EG_pd_d_), rather than estimating the trend in EG_pd_.

Note EG of vegetation phenology can also be influenced by phenology temperature sensitivity^34^. We therefore also calculated the elevational gradient of the temperature sensitivity of vegetation phenology. The elevational gradient of the temperature sensitivity of SOS was calculated as the following steps. (i) For each pixel, the preseason was determined as the period before SOS for which the absolute value of the correlation coefficient between SOS and air temperature was highest^63^. Note that since the relationship between SOS and temperature is usually regarded as negative, respectively, we thus exclude those positive correlation coefficients for SOS. (ii) For each 9 × 9 moving spatial window, we used an identical preseason length, which is the median values of preseason length of each grid. (iii) The temperature sensitivity of SOS was calculated using a linear regression for the period 1982–2011 with temperature as the dependent variable and SOS as the independent variable. (iv) The elevational gradient of temperature sensitivity of SOS was estimated for each 9 × 9 moving window. To examine the dominant factor driving the temporal change of EG_SOS_, we classified all grid into another four categories (categories a-d in Supplementary Table 1) based on the sign of EG_SOS_ trends, spring EG_tem_ trends, and elevation gradients of temperature sensitivity of SOS: (a) the EG_SOS_ trend is co-determined by both EG_tem_ and the temperature sensitivity of SOS, (b) the EG_SOS_ trend is primarily driven by the temperature sensitivity of SOS, (c) the EG_SOS_ trend is primarily driven by spring EG_tem_, (d) the EG_SOS_ trend is possibly driven by other factors.

### Reporting summary

Further information on research design is available in the Nature Research Reporting Summary linked to this article.

## Supplementary information

Supplementary Information

Reporting Summary

## Data Availability

The AVHRR GIMMS NDVI_3g_ datasets are available at https://ecocast.arc.nasa.gov/data/pub/gimms/3g.v1/. The Worldclim version 2 datasets can be accessed through website http://worldclim.org/version2. The ERA Interim datasets can be downloaded from website https://www.ecmwf.int/en/forecasts/datasets/reanalysis-datasets/era-interim. The Gridded Population of the World version 4 datasets (GPW v4) are available at http://sedac.ciesin.columbia.edu/data/collection/gpw-v4. The Night light data can be downloaded from https://www.ngdc.noaa.gov/eog/dmsp/downloadV4composites.html. The PEP725 database is available at http://www.pep725.eu/. All the relevant data from this study are also available from the corresponding author upon request.

## Code availability

All computer codes for the analysis of the data are available from the corresponding author on reasonable request.
