# Supplementary material for: Divergent changes in the elevational gradient of vegetation activities over the last 30 years
Source: Nat Commun. 2019 Jul 5;10:2970. doi: 10.1038/s41467-019-11035-w (PMC6611807; doi:10.1038/s41467-019-11035-w)
Supplement: Supplementary file 1 — Supplementary Information [file 41467_2019_11035_MOESM1_ESM.pdf]

Supplementary Information

Divergent changes in the elevational gradient of vegetation activities over the last 30 years

Gao et al.

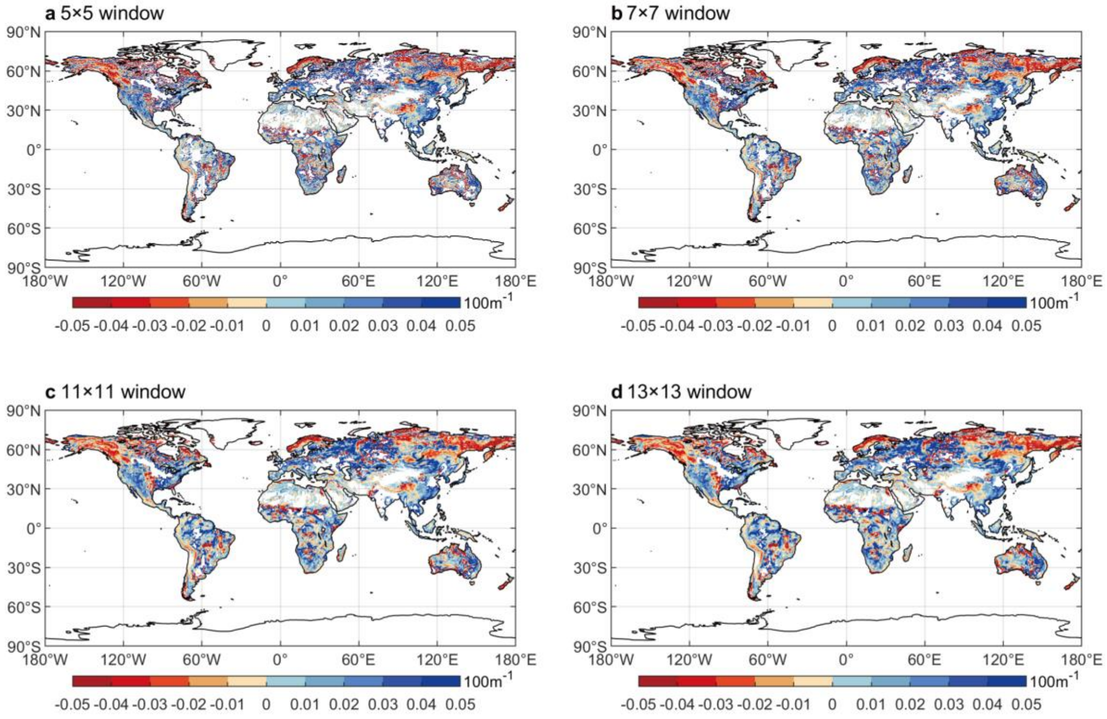

Supplementary Fig. 1 The elevational gradient of NDVI$_{max3}$ (EG$_{ndvimax}$) from 1982 to 2015. The same as **Fig. 1a**, but using different sized moving windows: (**a**) 5×5 moving windows, (**b**) 7×7 moving windows, (**c**) 11×11 moving windows, (**d**) 13×13 moving windows.

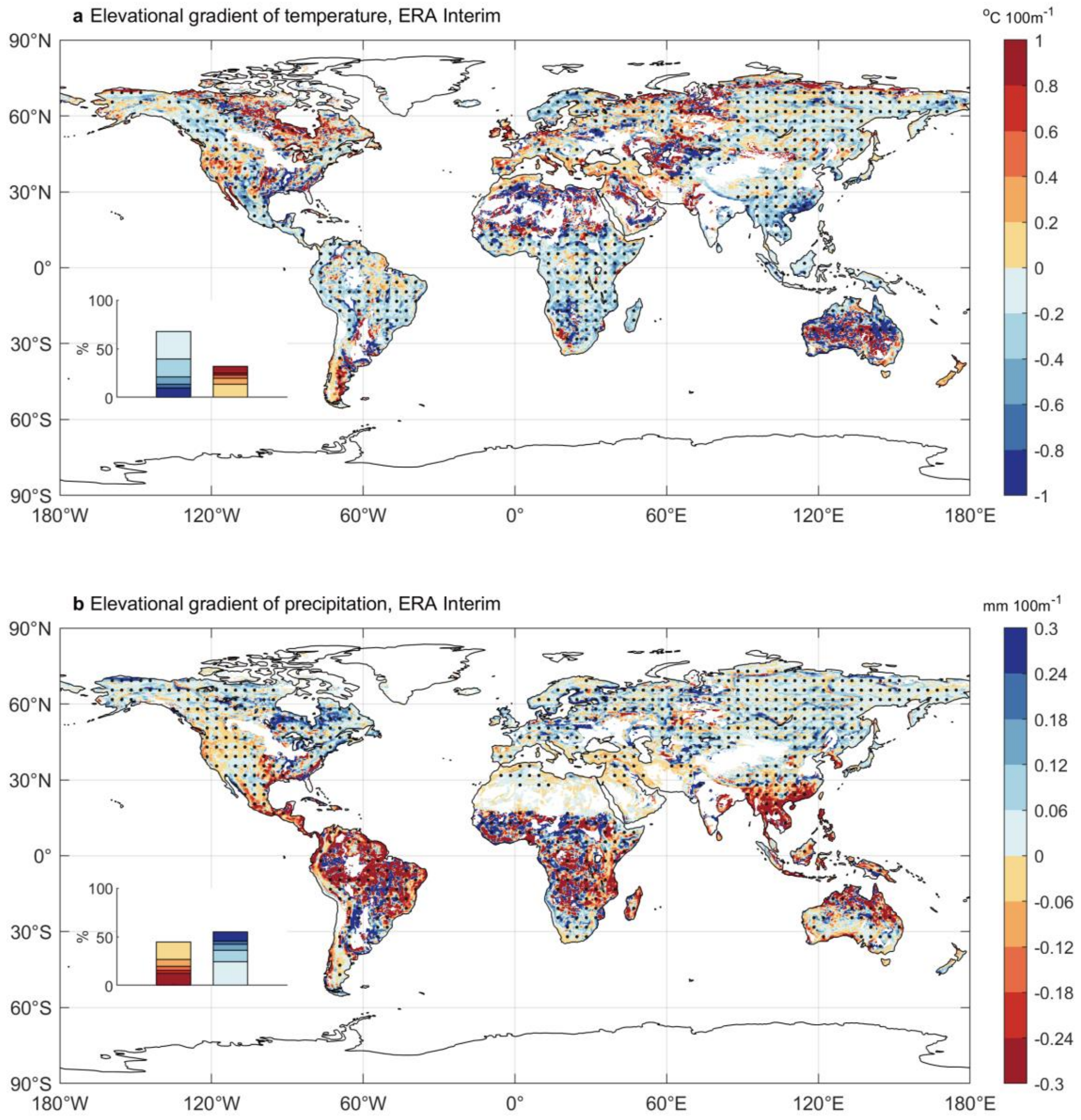

Supplementary Fig. 2 The elevational gradient (EG) of climate variables. Spatial patterns of the elevational gradient (EG) of (**a**) mean annual temperature and (**b**) average precipitation from the ERA Interim reanalysis dataset during 1982-2015. A frequency distribution of EG values is shown in the inset at the bottom-left for each panel. Regions marked with dots have statistically significant ($p<0.05$) EG values.

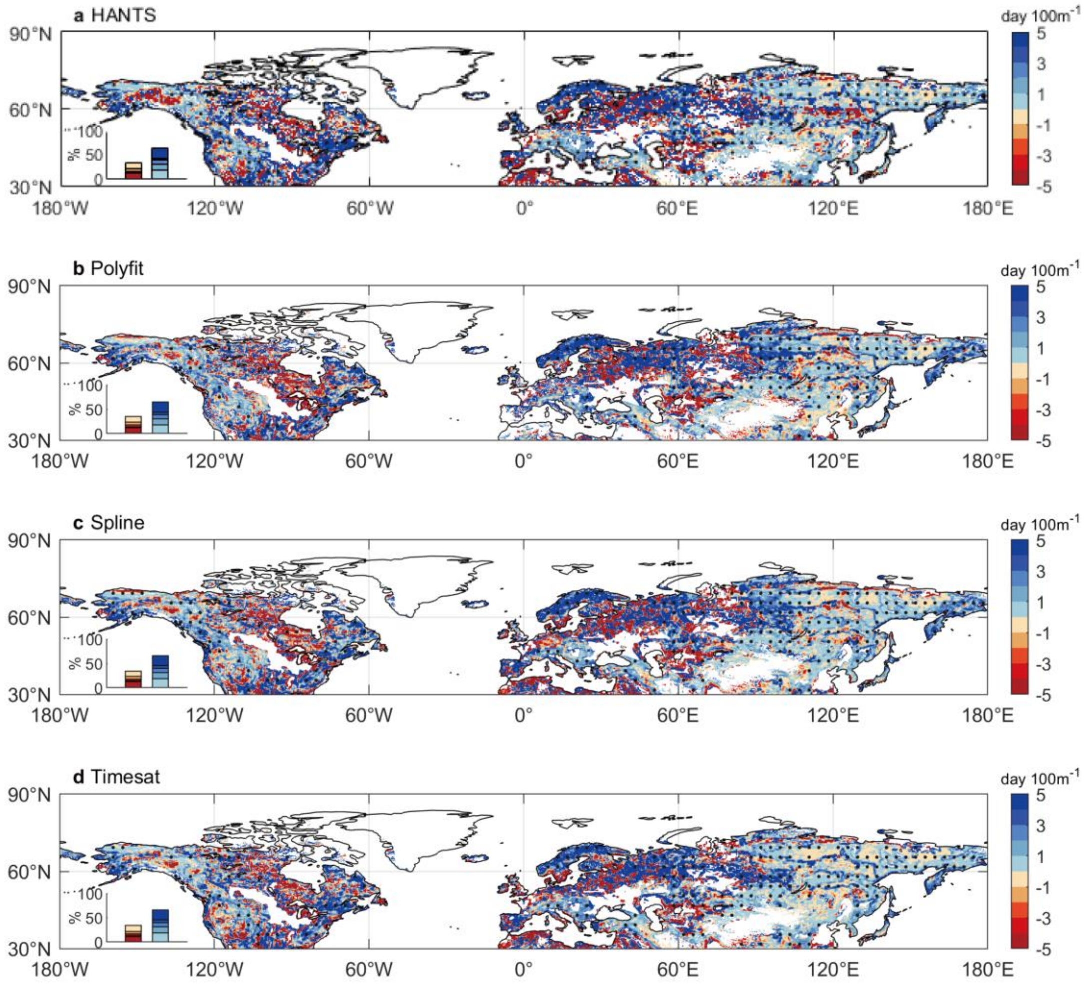

Supplementary Fig. 3 The spatial patterns of elevational gradient of SOS (EG_{SOS}). The same as **Fig. 1b**, but with SOS data estimated by each of the four different methods: (**a**) HANTS, (**b**) Polyfit, (**c**) Spline and (**d**) Timesat (see Methods). A frequency distribution of EG values is shown in the inset at the bottom-left for each panel. Regions marked with dots have statistically significant ($p<0.05$) EG values.

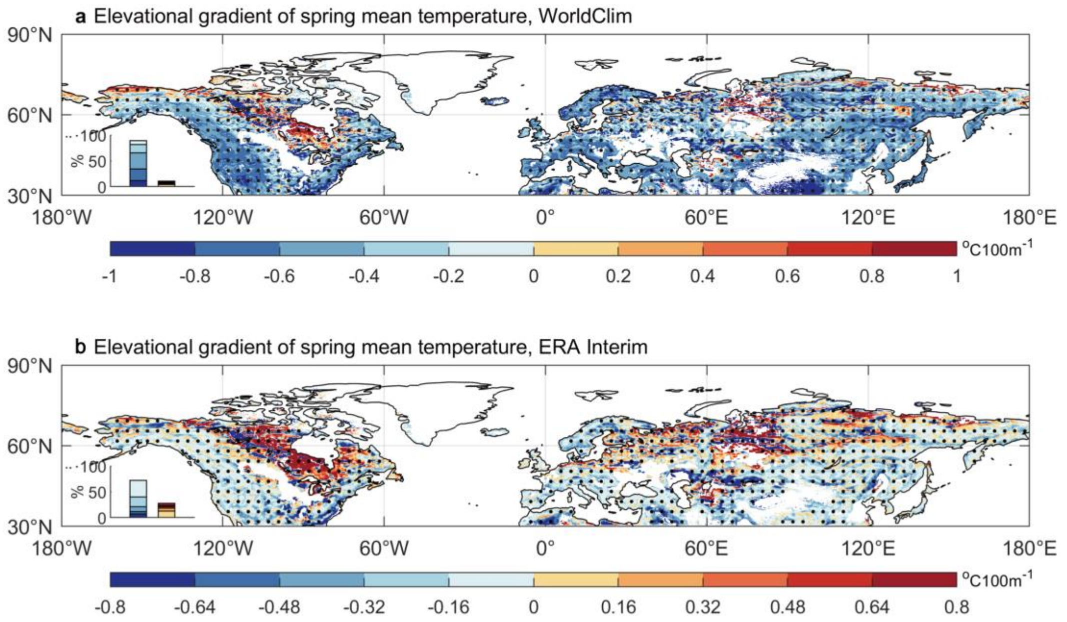

Supplementary Fig. 4 The elevational gradient of spring temperature. Spatial patterns of the elevational gradient (EG) of spring (March to May) mean temperature during 1982-2011 over the Northern Hemisphere (north of 30°N). The climate data were from the WorldClim dataset (**a**) and the ERA Interim reanalysis dataset (**b**). A frequency distribution of the EG values is shown in the inset at the bottom-left for each panel. Regions marked with dots have statistically significant ($p<0.05$) EG values.

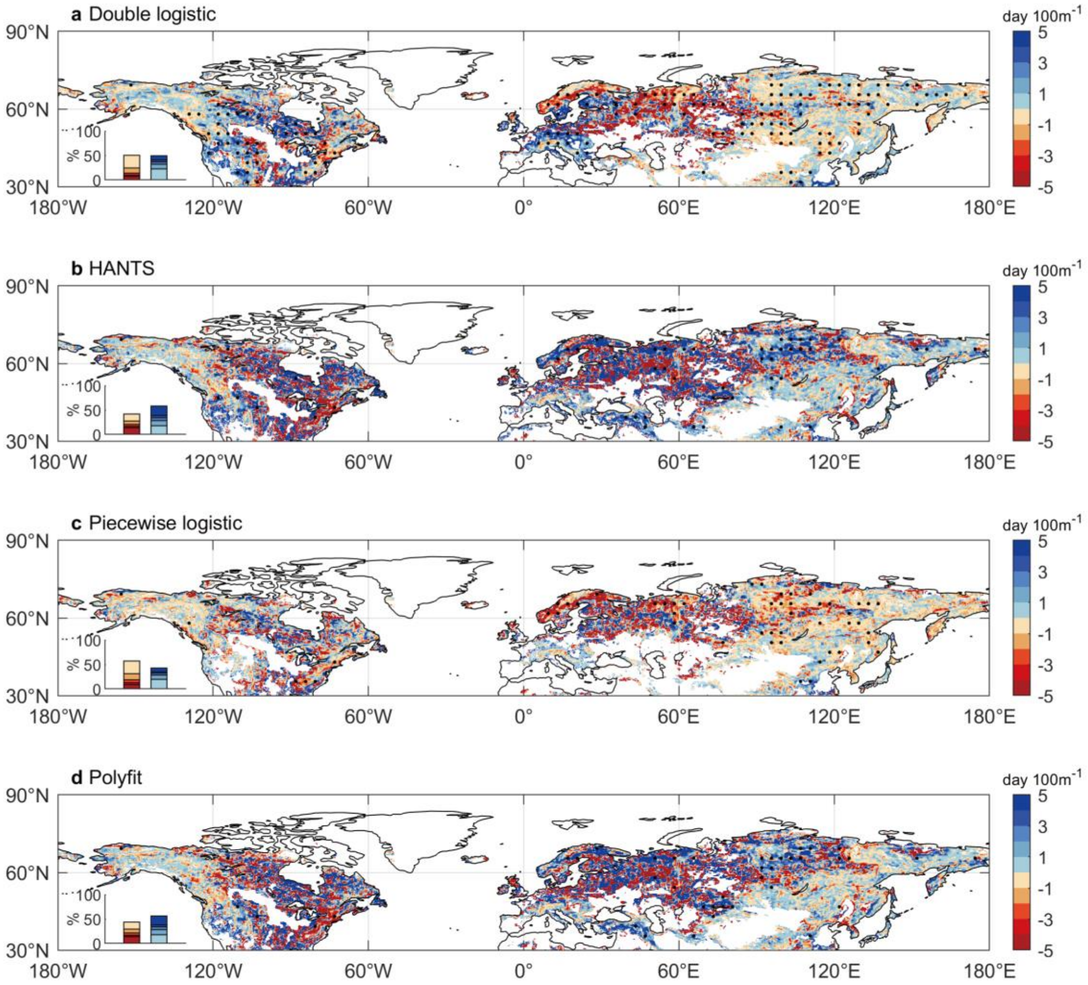

Supplementary Fig. 5 The spatial patterns of elevational gradient of EOS (EG_{EOS}). The same as **Fig. 1c**, but with EOS data estimated using each of the four different methods: (**a**) Double logistic, (**b**) HANTS, (**c**) Piecewise logistic and (**d**) Polyfit (see Methods). A frequency distribution of EG values is shown in the inset at the bottom-left for each panel. Regions marked with dots have statistically significant ($p<0.05$) EG values.

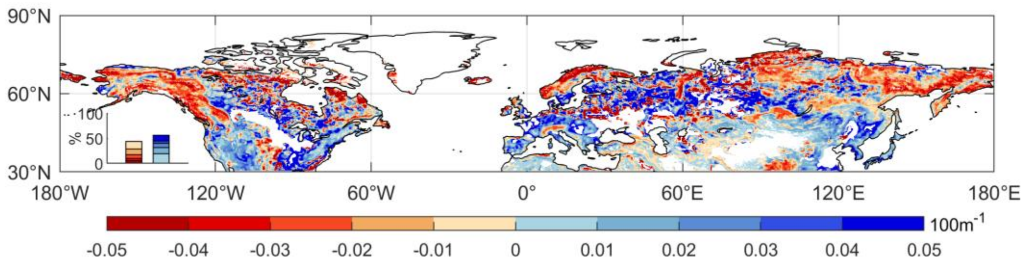

Supplementary Fig. 6 Spatial patterns of the elevational gradient for the mean autumn (September to November) NDVI during 1982-2015. A frequency distribution of EG values is shown in the inset at the bottom-left.

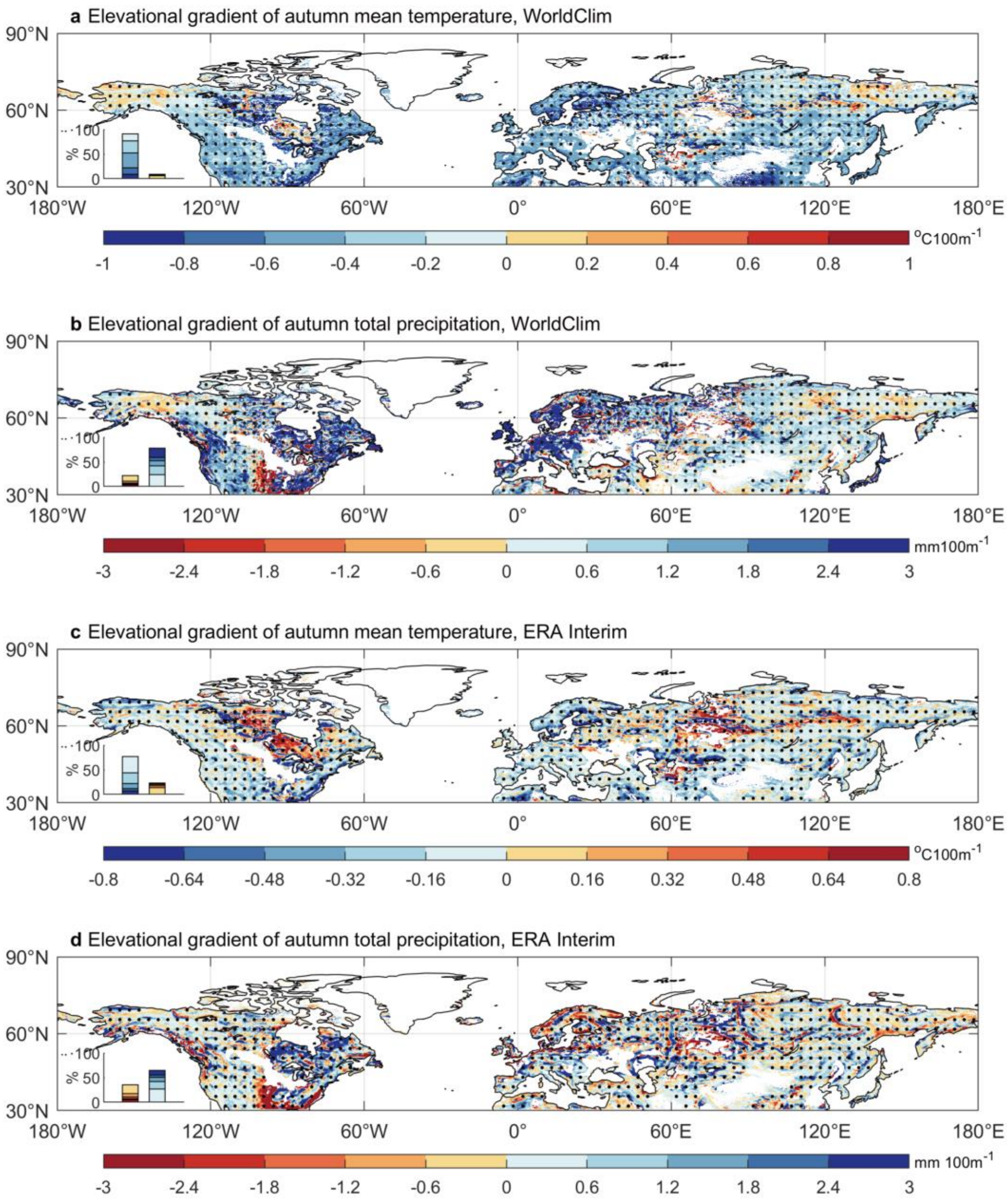

Supplementary Fig. 7 The elevational gradient of autumn temperature and precipitation. Spatial patterns of the elevational gradient of autumn (September to November) mean temperature (**a**, **c**) and total precipitation (**b**, **d**) during 1982-2011 over the Northern Hemisphere (north of 30°N). The climate data were from the WorldClim dataset (**a**, **b**) and the ERA Interim reanalysis dataset (**c**, **d**). A frequency distribution of EG values is shown in the inset at the bottom-left for each panel. Regions marked with dots have statistically significant ($p<0.05$) EG values.

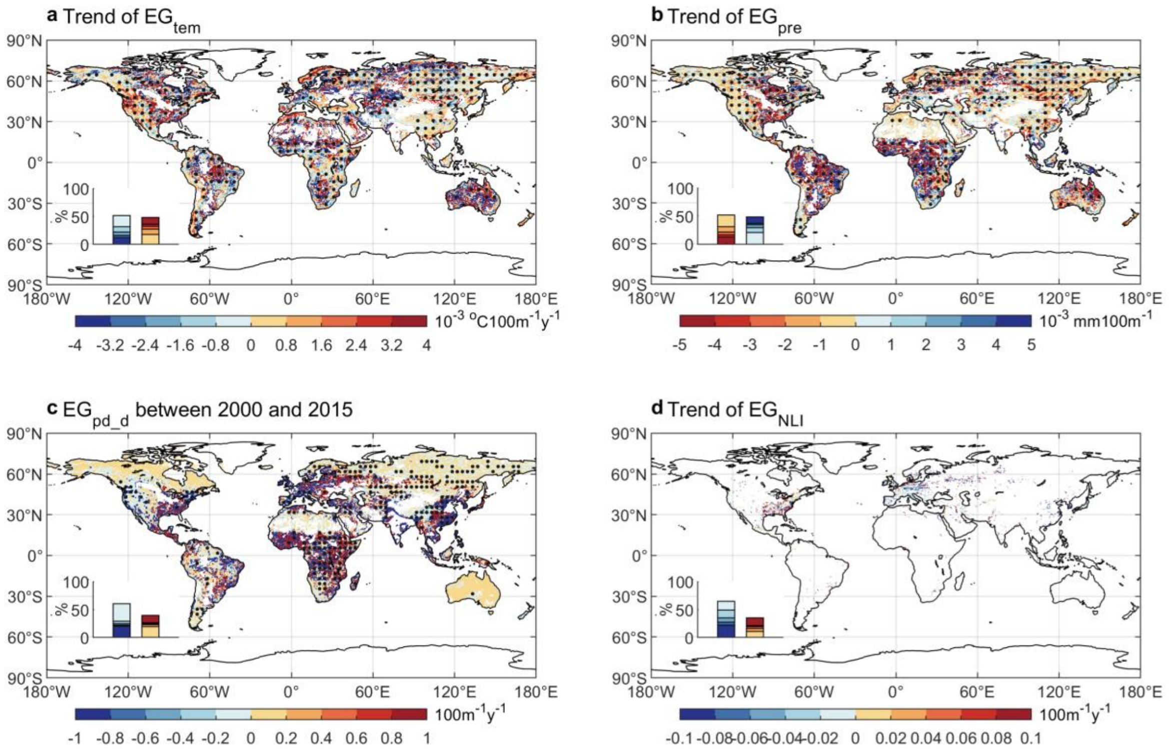

Supplementary Fig. 8 Spatial patterns of the linear temporal trend for the elevational gradients of climate variables and human activity indices. **a**, trend for the elevational gradient of mean annual temperature (EG_{tem}) during 1982 to 2015. **b**, trend for the elevational gradient of average precipitation (EG_{pre}) during 1982 to 2015. **c**, elevational gradient of the population density difference between 2015 and 2000 (EG_{pd_d}). **d**, trend for the elevational gradient of night light index (EG_{NLI}) during 1992 to 2013. Climate data was from ERA Interim dataset (see Methods). A frequency distribution of EG values is shown in the inset at the bottom-left for each panel. Regions marked with dots have statistically significant ($p<0.05$) EG values.

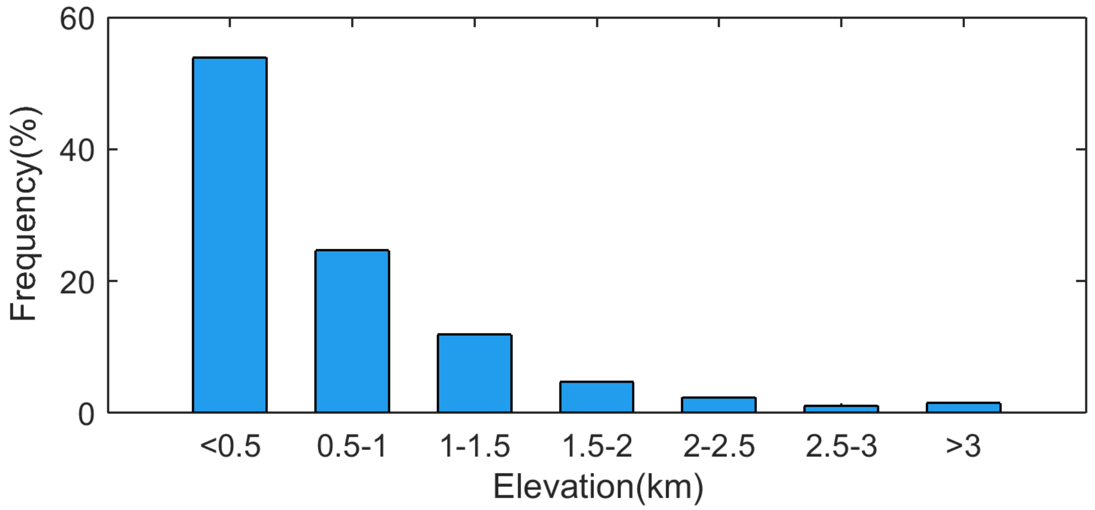

Supplementary Fig. 9 The frequency distribution of afforestation areas in China at different elevations based on the forest plantation map (2004-2008) provided by Shushi Peng[1].

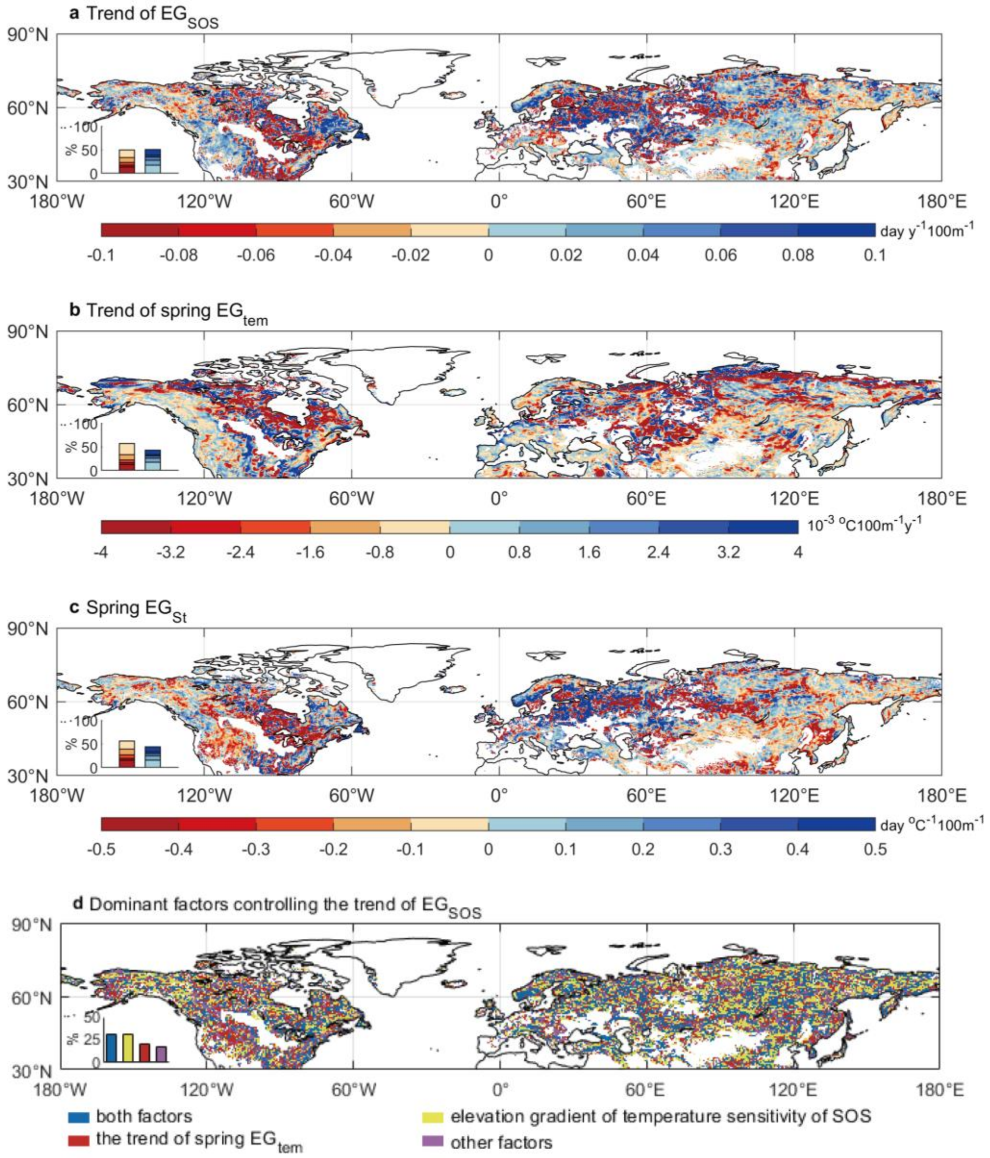

Supplementary Fig. 10 The trend of EG$_{SOS}$ and its driving factors. a, spatial pattern of the linear trend of EG$_{SOS}$ during 1982 to 2011. **b**, spatial pattern of the linear trend of the elevational gradients of spring (March to May) mean temperature (spring EG$_{tem}$) during 1982 to 2011. A frequency distribution of the trend values is shown in the inset at the bottom-left for panels (**a**) and (**b**). **c**, spatial pattern of the elevational gradient of temperature sensitivity of SOS (see Methods). A frequency distribution of the EG values is shown in the inset at the bottom-left for panel (**c**). **d**, spatial pattern of dominant factors controlling the trend of EG$_{SOS}$ (see Methods). A frequency distribution of grids controlling by different factors is shown in the inset at the bottom-left of panel (**d**).

Supplementary Table 1. Scenarios of temperature and temperature sensitivity in controlling the trend of EG_{SOS}

Category	Temporal trend of EG_{SOS}	Elevational gradient of temperature sensitivity of SOS	Temporal trend of spring EG_{tem}
(a)	+	+	-
	-	-	+
(b)	+	+	+
	-	-	-
(c)	+	-	-
	-	+	+
(d)	+	-	+
	-	+	-

Supplementary Reference

1. Peng, S.-S. *et al.* Afforestation in China cools local land surface temperature. *Proc. Natl. Acad. Sci.* **111**, 2915–2919 (2014).
